# Supplementary material for: Protection against SARS-CoV-2 Omicron BA.4/5 variant following booster vaccination or breakthrough infection in the UK
Source: Nat Commun. 2023 May 16;14:2799. doi: 10.1038/s41467-023-38275-1 (PMC10187514; doi:10.1038/s41467-023-38275-1)
Supplement: Supplementary file 1 — Supplementary Information [file 41467_2023_38275_MOESM1_ESM.pdf]

Supplementary information for

Protection against SARS-CoV-2 Omicron BA.4/5 variant following booster vaccination or breakthrough infection in the UK

Jia Wei, Philippa C. Matthews, Nicole Stoesser, John N Newton, Ian Diamond, Ruth Studley, Nick Taylor, John I Bell, Jeremy Farrar, Jaison Kolenchery, Brian D. Marsden, Sarah Hoosdally, E Yvonne Jones, David I Stuart, Derrick W. Crook, Tim E. A. Peto, A. Sarah Walker, Koen B. Pouwels, David W. Eyre and the COVID-19 Infection Survey team

Contents

- **Supplementary Figures 1-12**
- **Supplementary Tables 1-7**

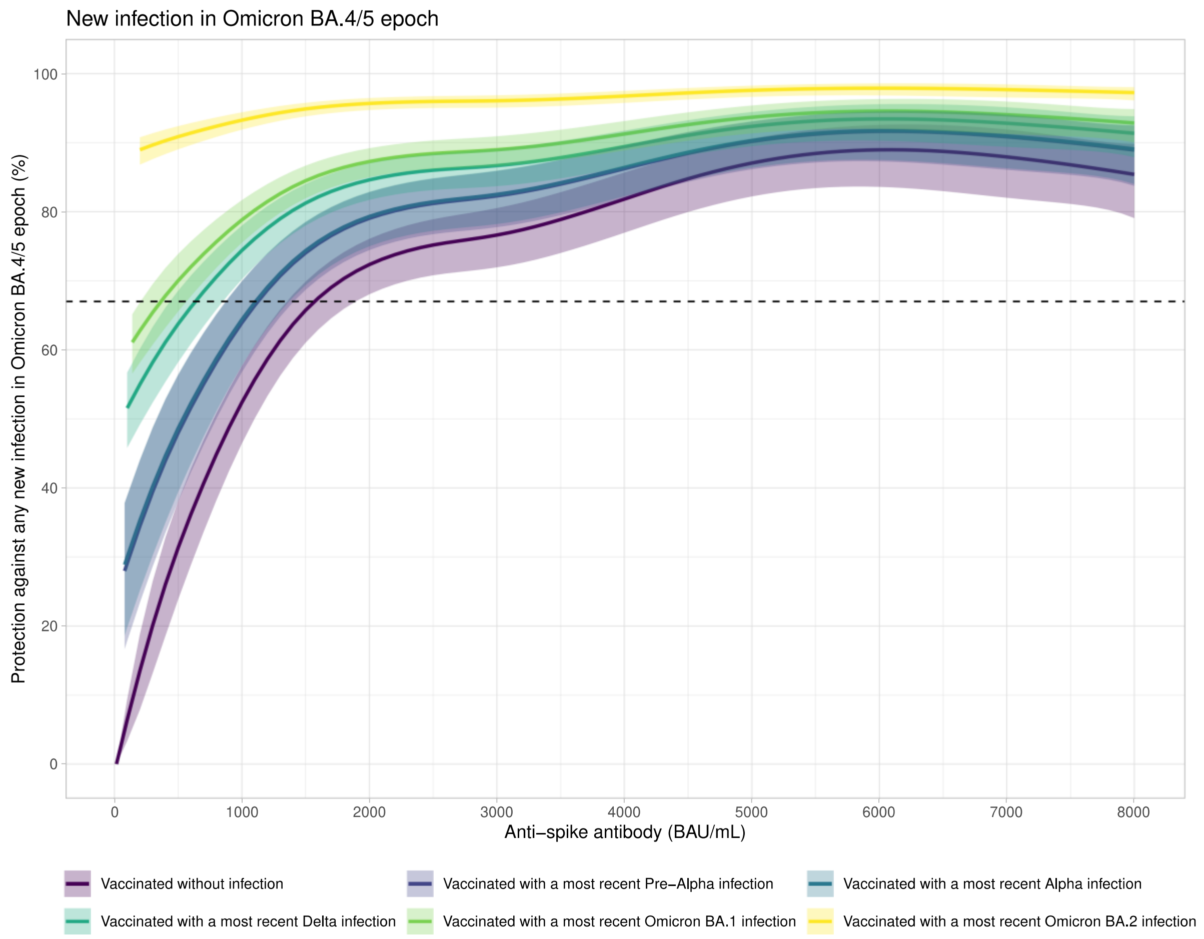

Supplementary Fig. 1. Association between anti-spike IgG levels and mean protection from new SARS-CoV-2 infection in the Omicron BA.4/5 epoch using the most recent antibody measurement obtained 21–59 days before the current assessment by infection variant. The 95% CIs are calculated by prediction ± 1.96 × standard error of the prediction. Participants with 1 (629 assessments, 0.3%), 2 (7,657 assessments, 3.7%), 3 (171,650 assessments, 83.9%), or 4 (24,753 assessments, 12.1%) vaccinations were grouped together. Protection is defined as relative protection against a baseline protection afforded by 16 BAU/mL in those vaccinated without infection, which is the threshold for vaccine non-responders (see Methods). Antibody measurements were plotted after the 1% percentile in each group (16, 80, 80, 100, 140, 200 BAU/mL, respectively). From the plot, estimates for Pre-Alpha and Alpha infection are similar (lines overlapped). Therefore, Pre-Alpha and Alpha infection are combined in the main model to increase power.

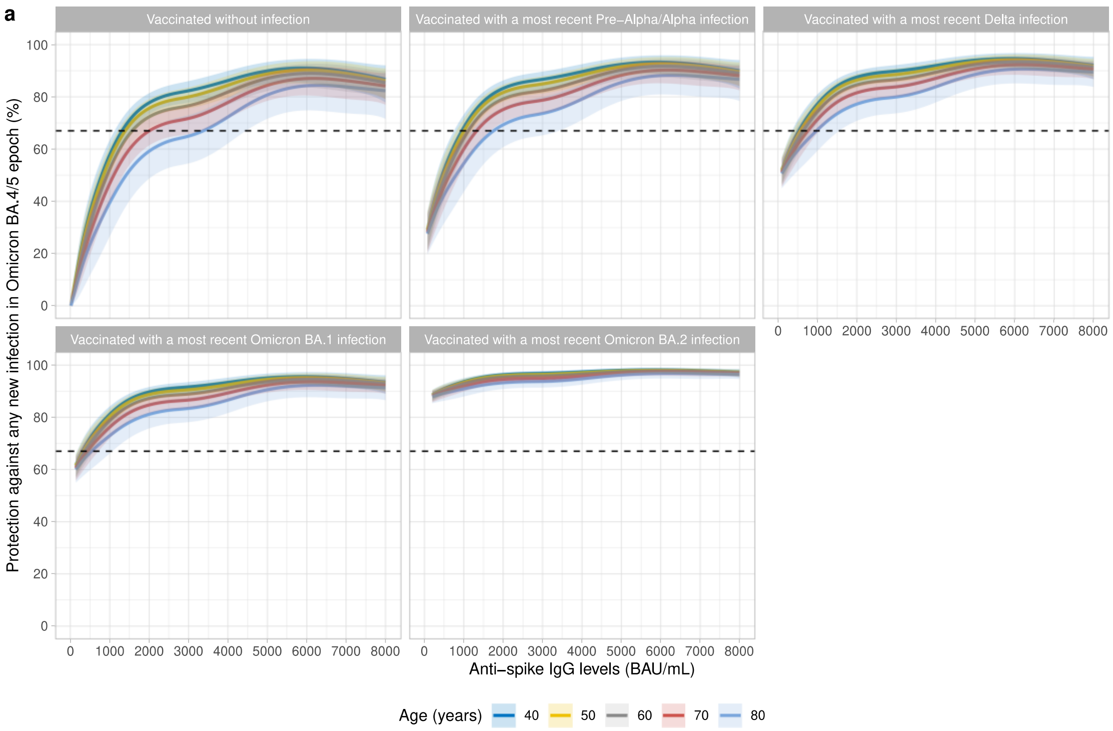

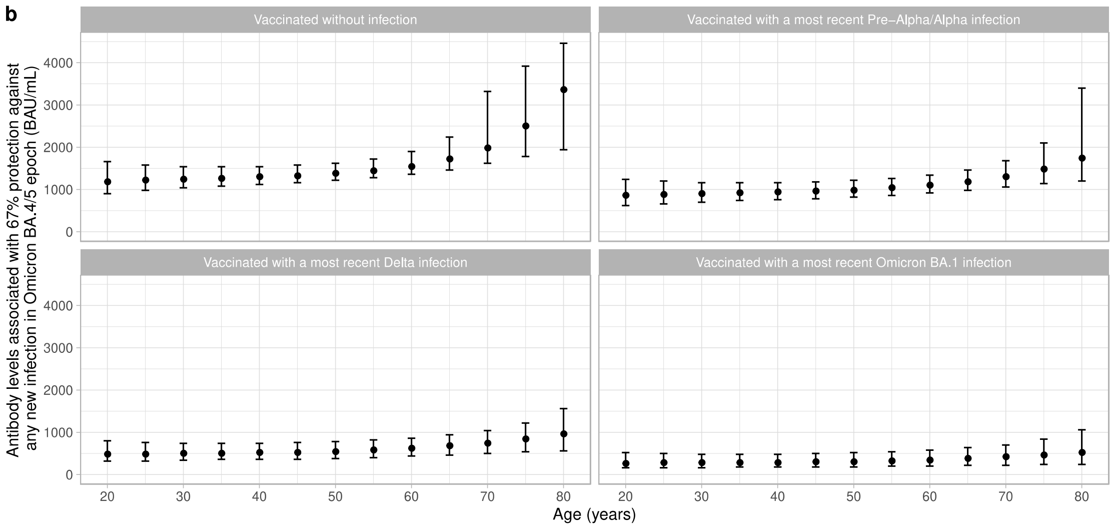

Supplementary Fig. 2. Effect of age on protection from new infection in the Omicron BA.4/5 epoch afforded by different antibody levels. A, Association between anti-spike IgG levels and mean protection from SARS-CoV-2 infection by age. Colour represents individual ages. Dotted line represents 67% protection against a baseline protection afforded by 16 BAU/mL in those vaccinated without infection, which is the threshold for vaccine non-responders. Five groups are investigated: vaccinated participants without evidence of prior infection, vaccinated participants with a most recent Pre-Alpha or Alpha infection, vaccinated participants with a most recent Delta infection, vaccinated participants with a most recent Omicron BA.1 infection, and vaccinated participants with a most recent Omicron BA.2 infection. Participants with 1 (629 assessments, 0.3%), 2 (7,657 assessments, 3.7%), 3 (171,650 assessments, 83.9%), or 4 (24,753 assessments, 12.1%) vaccinations were grouped together. Antibody measurements were plotted after the 1st percentile overall in each previous infection group (16, 80, 100, 140, 200 BAU/mL, respectively). **b,** comparison of mean antibody levels associated with 67% protection against infection by age, based on N=106,653, 13,009, 15,745 and 26,736 visits in the four groups. The 95% CIs are calculated by prediction ± 1.96 × standard error of the prediction.

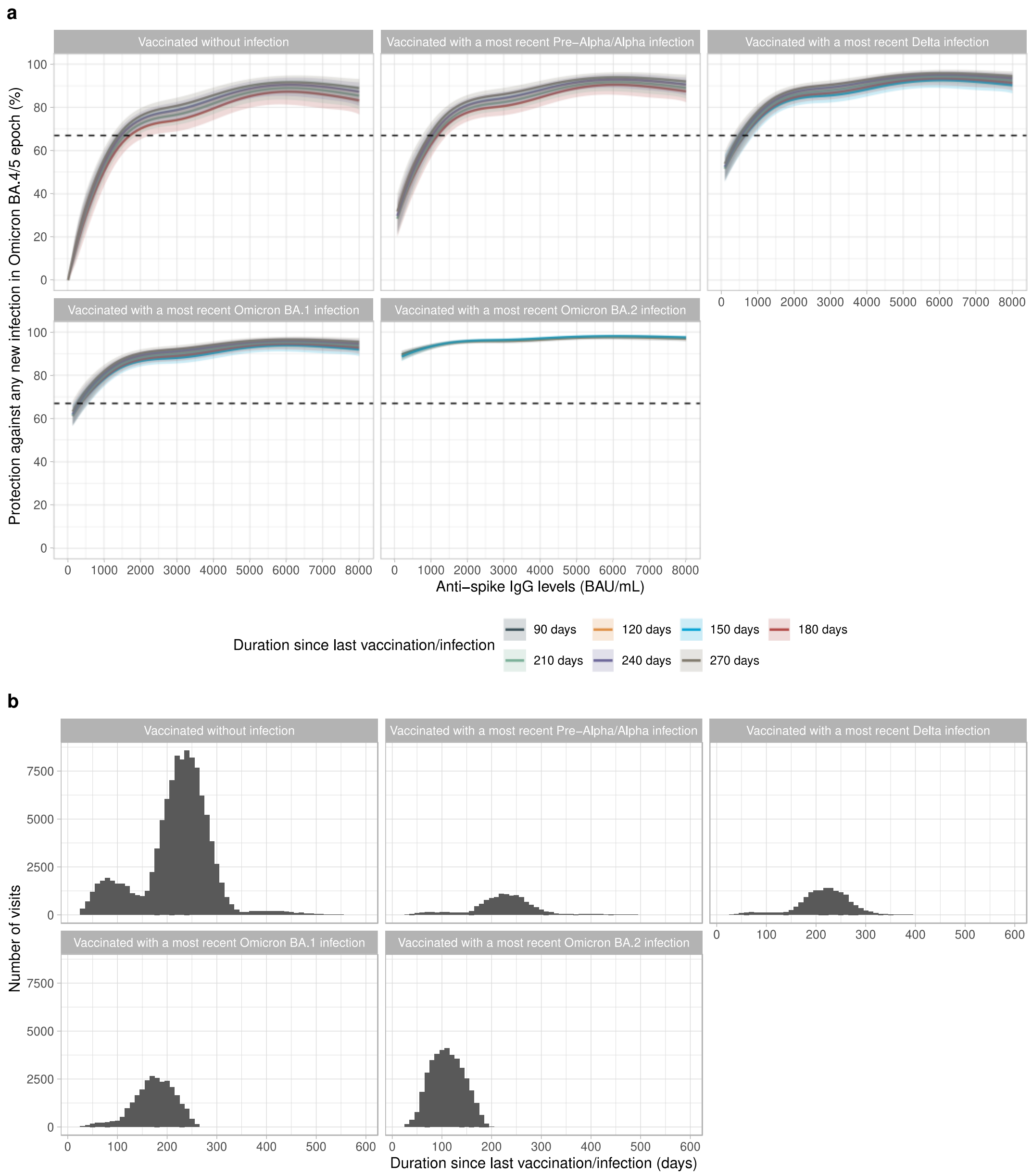

Supplementary Fig. 3. Effects from time since last vaccination or infection on protection from new infection in the Omicron BA.4/5 epoch afforded by different antibody levels. a, Association between anti-spike IgG levels and mean protection from SARS-CoV-2 infection by duration since last vaccination or infection to the current assessment. Colour represents duration since last vaccination or infection. Dotted line represents 67% protection against a baseline protection from 16 BAU/mL in those vaccinated without infection, which is the threshold for vaccine non-responders. Five groups are investigated: vaccinated participants without evidence of prior infection, vaccinated participants with a most recent Pre-Alpha or Alpha infection, vaccinated participants with a most recent Delta infection, vaccinated participants with a most recent Omicron BA.1 infection, and vaccinated participants with a most recent Omicron BA.2 infection. Participants with 1 (629 assessments, 0.3%), 2 (7,657 assessments, 3.7%), 3 (171,650 assessments, 83.9%), or 4 (24,753 assessments, 12.1%) vaccinations were grouped together. Antibody measurements were plotted after the 1st percentile overall in each previous infection group (16, 80, 100, 140, 200 BAU/mL, respectively). The 95% CIs are calculated by prediction ± 1.96 × standard error of the prediction. **b,** Distribution of duration from last vaccination or infection to the current assessment by group. The median duration was 230, 230, 220, 177, 110 days for the five groups.

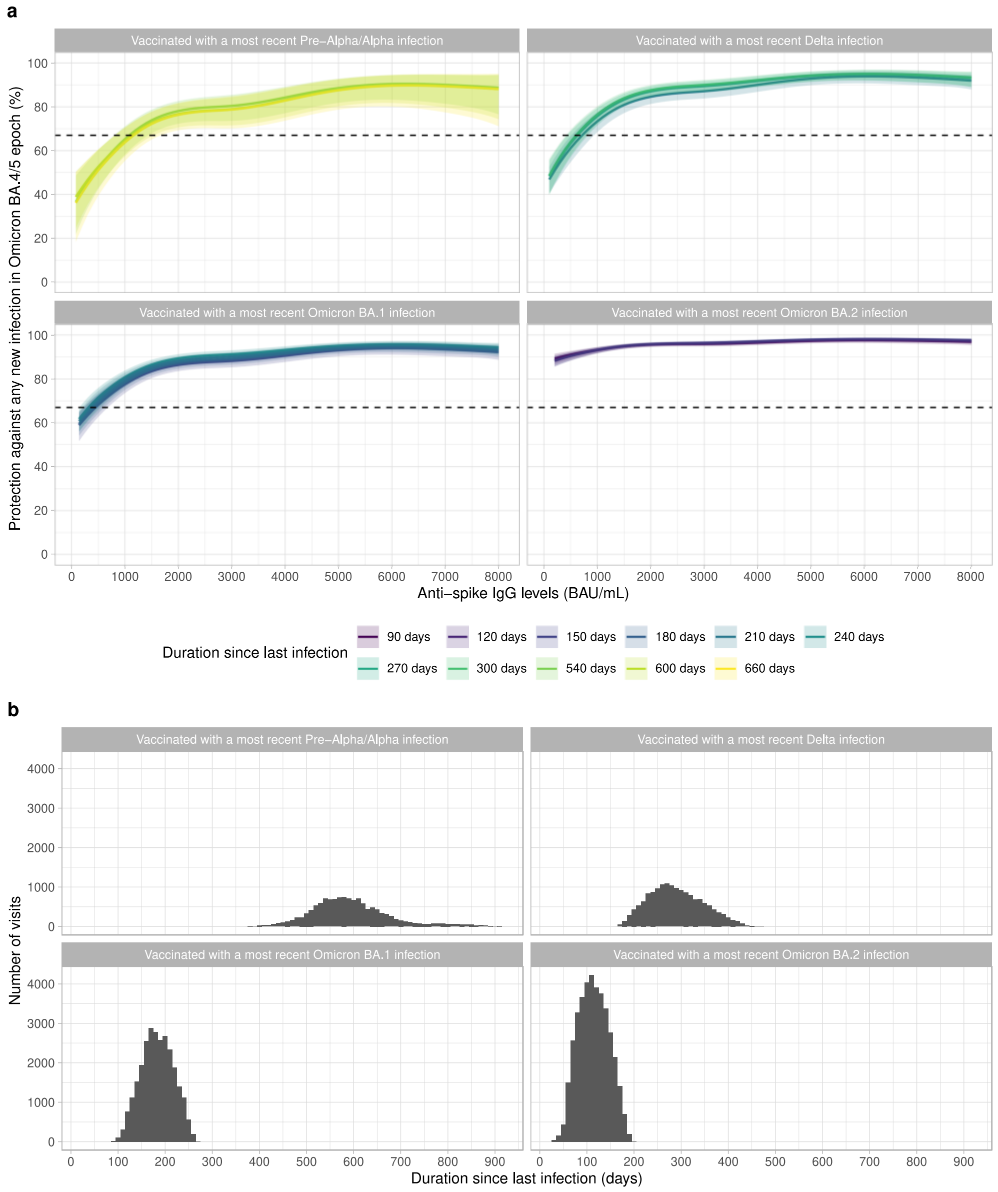

Supplementary Fig. 4. Effects from time since last infection on protection from new infection in the Omicron BA.4/5 epoch afforded by different antibody levels. a, Association between anti-spike IgG levels and mean protection from SARS-CoV-2 infection by duration since last infection to the current assessment, ignoring time from last vaccination in a separate model. Colour represents duration since last infection. Dotted line represents 67% protection against a baseline protection from 16 BAU/mL in those vaccinated without infection, which is the threshold for vaccine non-responders. Five groups are investigated: vaccinated participants without evidence of prior infection, vaccinated participants with a most recent Pre-Alpha or Alpha infection, vaccinated participants with a most recent Delta infection, vaccinated participants with a most recent Omicron BA.1 infection, and vaccinated participants with a most recent Omicron BA.2 infection. Only the four infected groups are shown here to show the effects of time since last infection. Participants with 1 (629 assessments, 0.3%), 2 (7,657 assessments, 3.7%), 3 (171,650 assessments, 83.9%), or 4 (24,753 assessments, 12.1%) vaccinations were grouped together. Antibody measurements were plotted after the 1st percentile overall in each previous infection group (80, 100, 140, 200 BAU/mL, respectively). The 95% CIs are calculated by prediction ± 1.96 × standard error of the prediction. **b,** Distribution of duration from last infection to the current assessment by group. The median duration was 585, 185, 183, 113 days for the four groups.

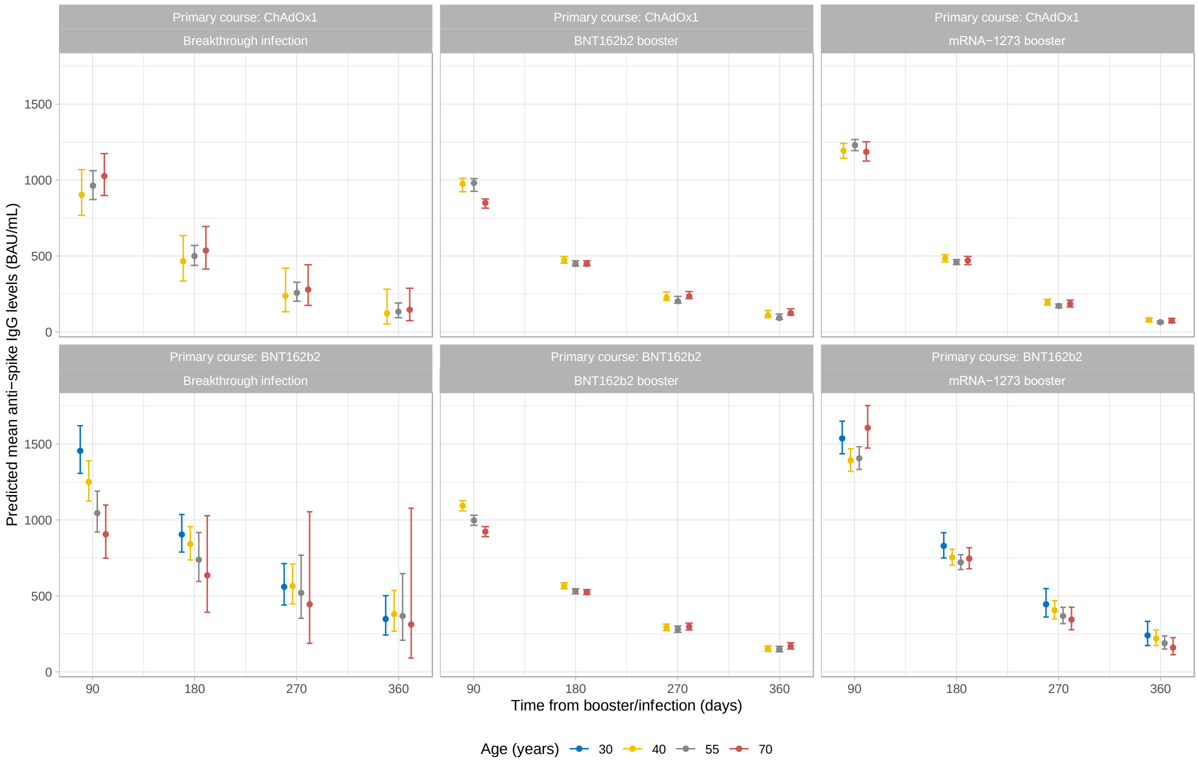

Supplementary Fig. 5. Predicted mean anti-spike IgG levels (95%CrI) at 90, 180, 270, and 360 days after the third/booster vaccination or breakthrough infection by ages. 30 years only estimated for those who had BNT162b2 as primary and were boosted by mRNA-1273 or infection due to low numbers in other groups. Numbers of participants in each panel are: ChAdOx1 primary course: N=4,214, 41,165, and 14,748; BNT162b2 primary course: N=1,857, 24,767, and 4,408 for breakthrough infection, BNT162b2 booster, and mRNA-1273 booster, respectively.

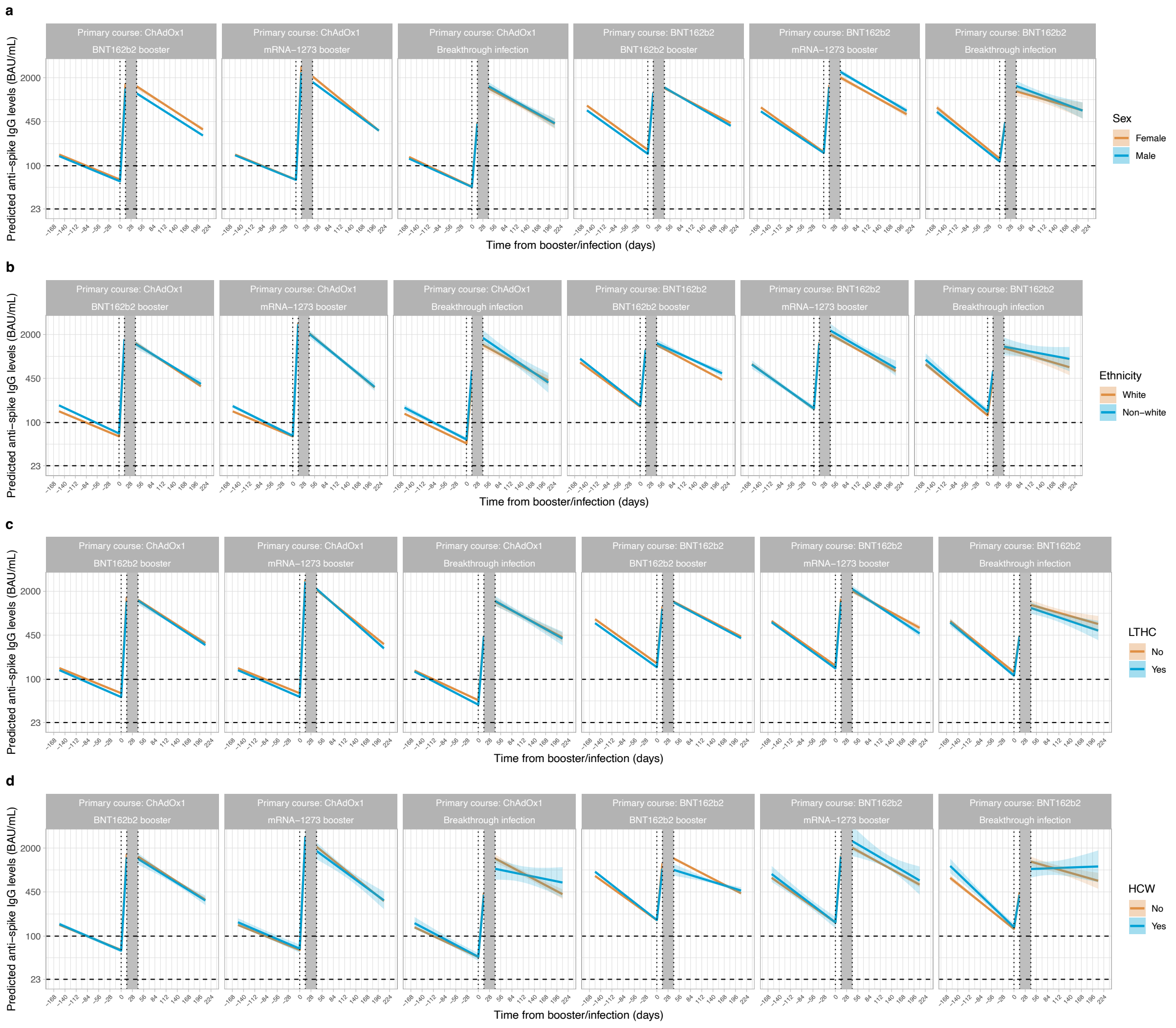

Supplementary Fig. 6. Posterior predicted trajectories of mean anti-spike IgG levels (95%CrI) from third/booster vaccination or infection by sex, ethnicity, long-term health condition, and healthcare role. Models are adjusted for age, sex, ethnicity, time from second vaccination to booster/infection, long-term health conditions, and healthcare role. Plotted at the reference categories (55y, 6 months from second vaccination to booster/infection, female, white ethnicity, not reporting long-term health condition, not working in healthcare). Plots are separated by primary vaccine course and booster types or infection. Predicted values are plotted on a log scale. Black dashed lines indicate the correlate for 67% protection against Delta variant (100 BAU/mL) and the threshold of IgG positivity (23 BAU/mL).

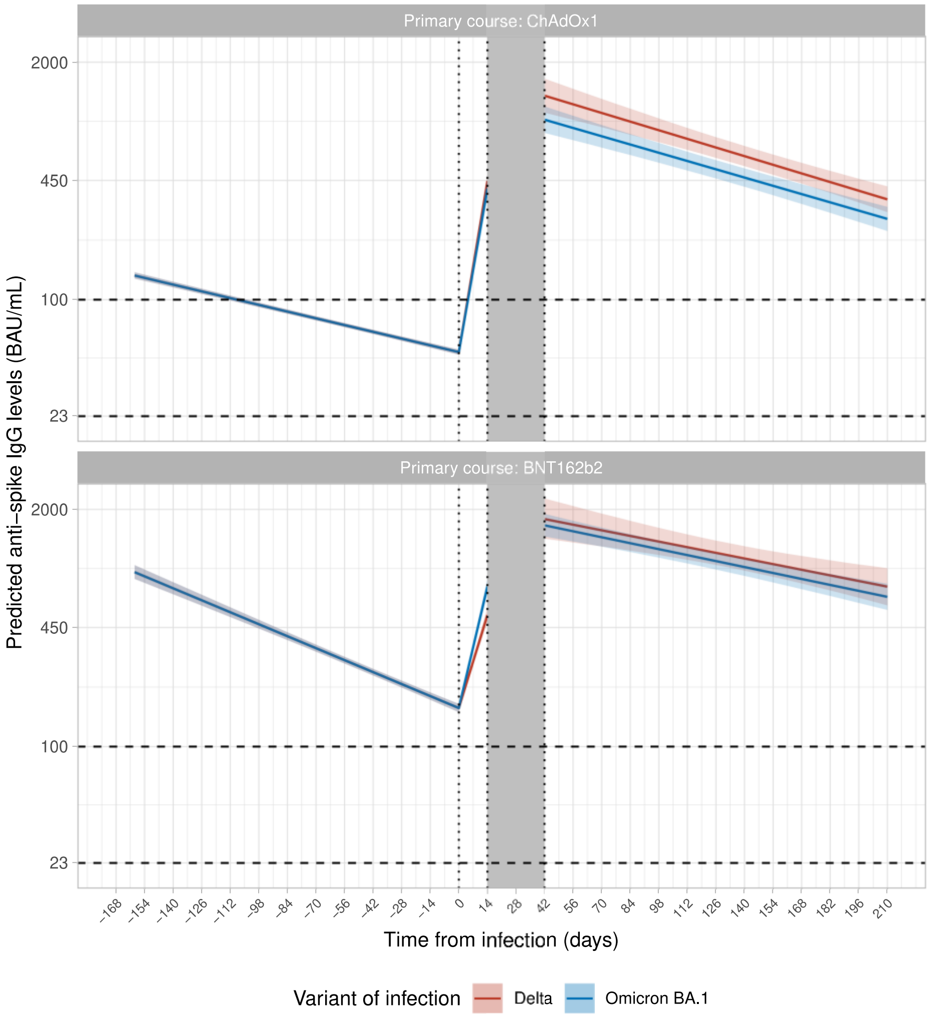

Supplementary Fig. 7. Posterior predicted trajectories of mean anti-spike IgG levels (95%CrI) after infection by SARS-CoV-2 variant (Delta vs Omicron BA.1). Plotted by primary vaccine course (ChAdOX1 or BNT162b2). Plotted at the reference categories (40y, 6 months from second vaccination to booster/infection, female, white ethnicity, not reporting long-term health condition, not working in healthcare). Black dashed lines indicate the correlate for 67% protection against Delta variant (100 BAU/mL) and the threshold of IgG positivity (23 BAU/mL). Shaded area between 14- and 42-days post infection represents different timepoints individuals reach peak antibody levels. No differences on antibody levels and subsequent decline by infection variant.

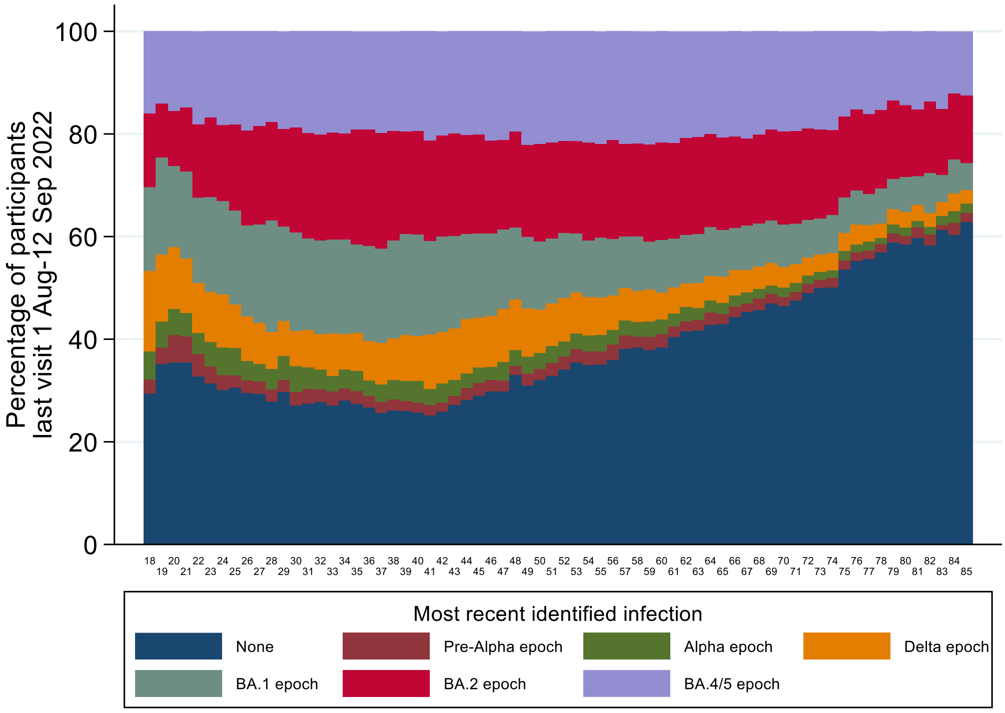

Supplementary Fig. 8. Percentages of participants' most recent previous infection by age using last assessment from 1 August 2022 to 12 September 2022.

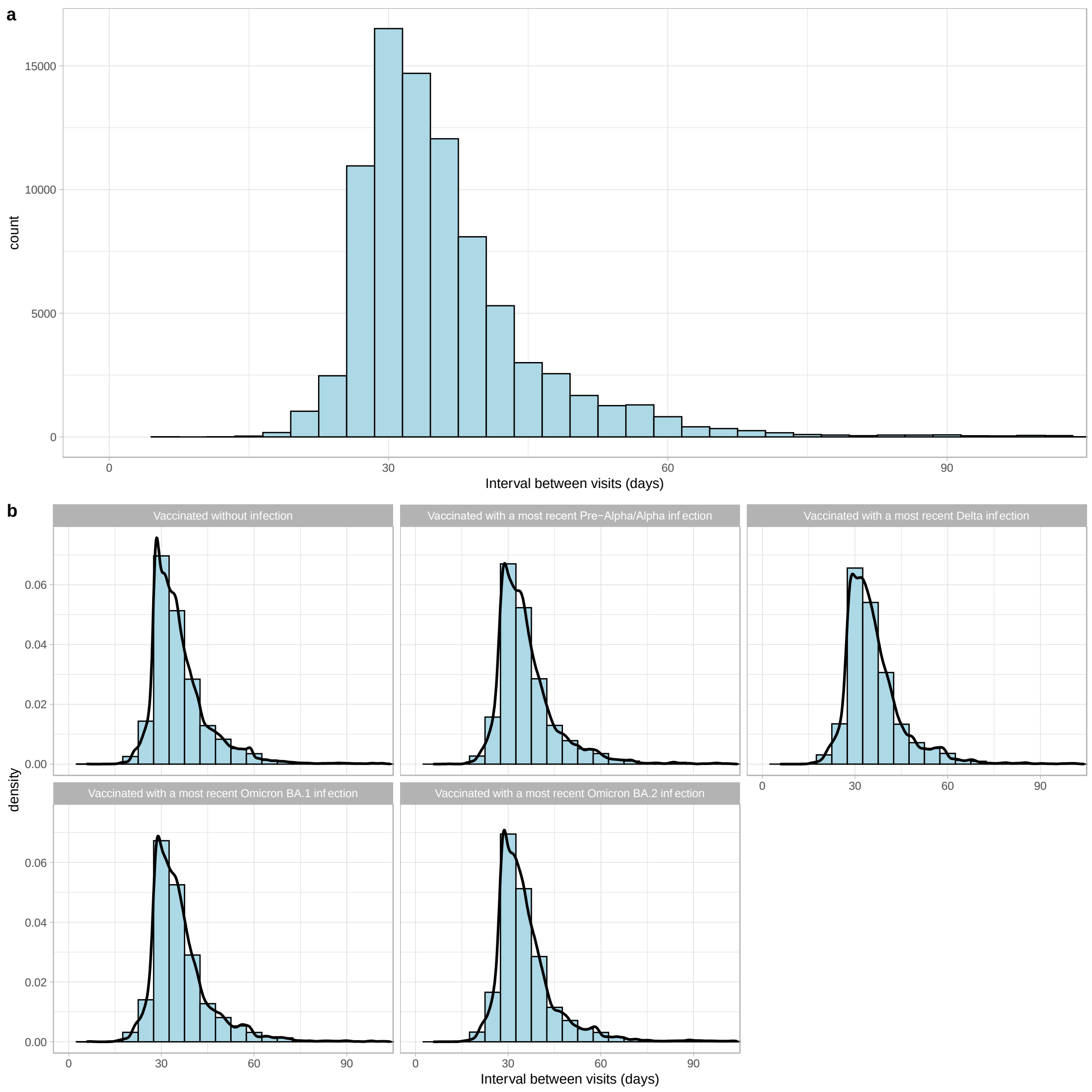

Supplementary Fig. 9. Distribution of the intervals between survey assessments included in the study. Assessments were planned on a 28–42-day cycle to achieve overall targets for swabs taken per month**.** Most intervals between assessments are <45 days, reducing the number of missed infections due to missed assessments (panel a). Additional information about positive tests taken outside the study was also used to classify previous infection status, see Methods. There was no evidence that this distribution varied by exposure groups (panel b).

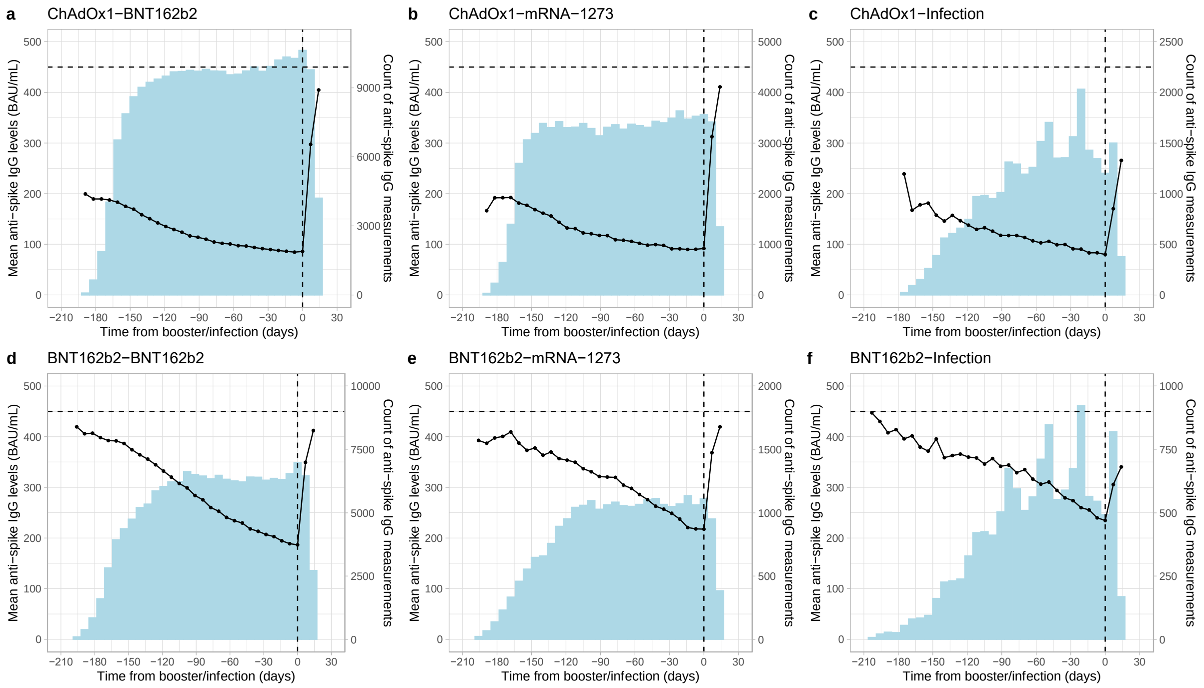

Supplementary Fig. 10. Mean anti-spike IgG levels (BAU/mL) and count of anti-spike IgG measurements by day from 21 days after the second vaccination to 14 days after the booster/infection. Panels are separated by primary vaccination course (ChAdOx1, BNT162b2) and boosting event (BNT162b2, mRNA-1273, breakthrough infection). Time 0 indicates the date participant receiving a booster vaccination or the first date of a breakthrough infection. Horizontal dotted line indicates the upper limit of quantification in 1:50 dilution, which is 450 BAU/mL. Measurements above the upper limit of quantification of 450 BAU/mL are considered to be >450 BAU/mL in analyses.

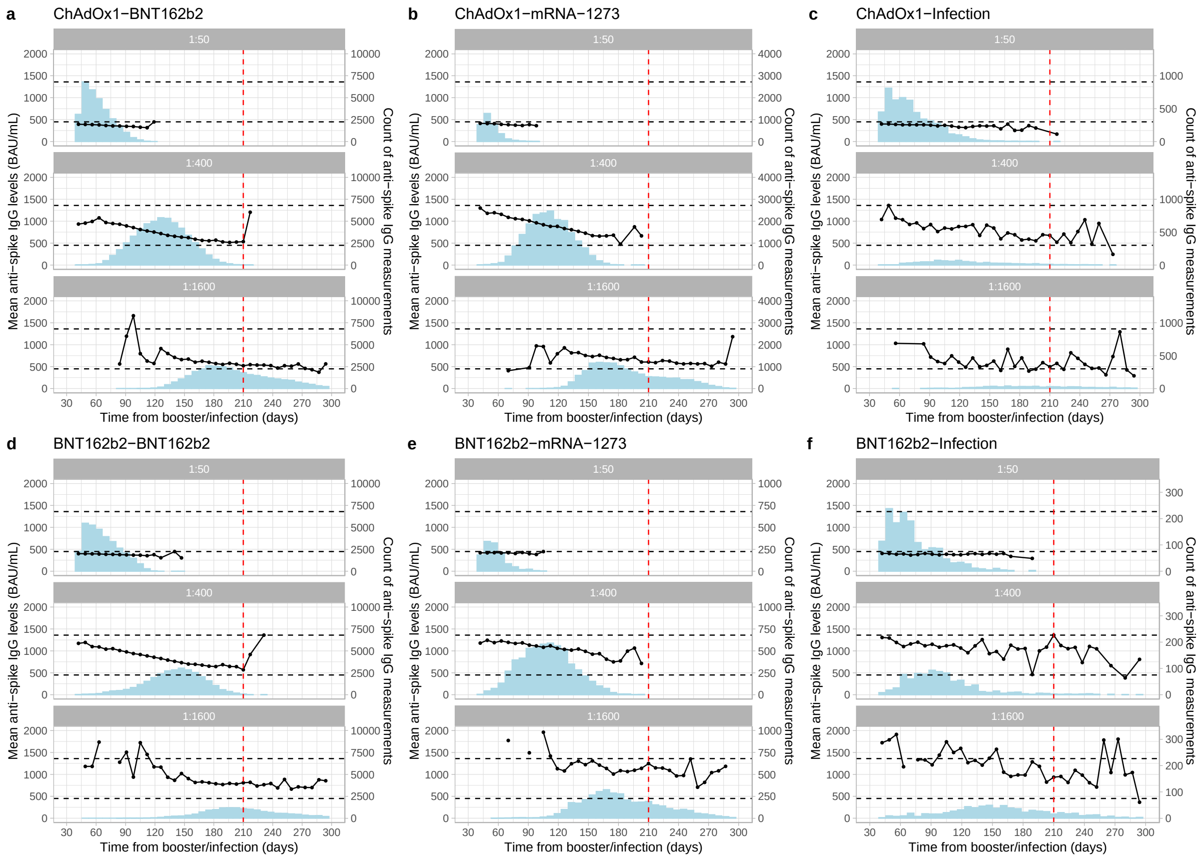

Supplementary Fig. 11. Mean anti-spike IgG levels (BAU/mL) and count of anti-spike IgG measurements by day from 42 days after the booster/infection for antibody decline modelling. Panels are separated by primary vaccination course (ChAdOx1, BNT162b2) and boosting event (BNT162b2, mRNA-1273, breakthrough infection). Measurements in different dilutions (1:50, 1:400, 1:1600) are plotted separately. Horizontal black dotted lines indicate the upper limits of quantification in 1:50 and 1:400 dilution, which are 450 and 1360 BAU/mL. Measurements above the upper limit of quantification of 450 BAU/mL in 1:50 dilution are considered to be >450 BAU/mL in analyses, and similarly for 1:400 and 1:1600 dilutions. We included data until 210 days after the booster/infection to ensure the same endpoint across groups (red dotted lines).

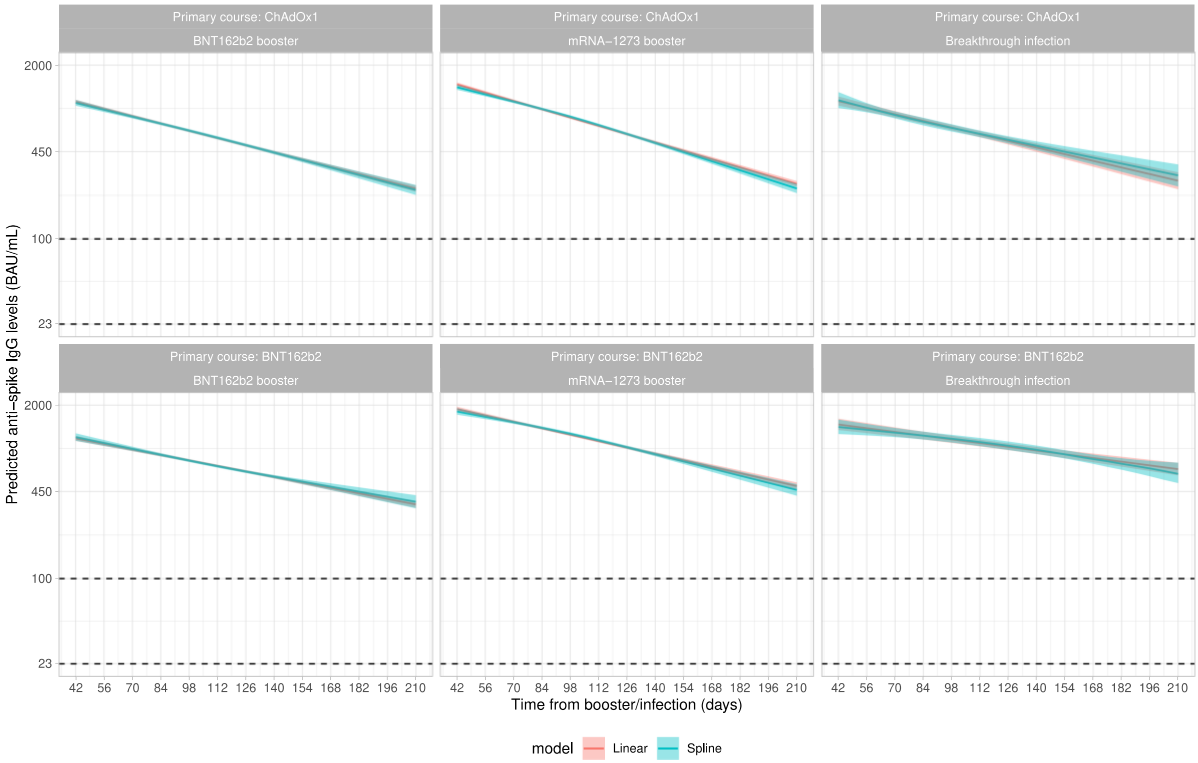

Supplementary Fig. 12. Comparison of linear exponential model with spline-based model in examining non-linearity of antibody decline. Y-axis represents posterior predicted mean trajectories (95%Crl) of anti-spike IgG levels plotted on a log scale. The estimated trajectory from the spline model (with 3 knots placed at 10th, 50th, and 90th of observed time points) is similar to the linear exponential model for all six groups, indicating that there was no evidence of antibody decline flattening over the period of observation (up to 210 days from booster/infection).

		Test-negative assessments	Test-positive assessments	%	Combined test-negative assessments	Combined test-positive assessments	%
**All infection**	Vaccinated without infection	100,973	5,680	5.3			
	Vaccinated with a most recent Pre-Alpha infection	5,730	215	3.6	12,536	473	3.6
	Vaccinated with a most recent Alpha infection	6,806	258	3.7			
	Vaccinated with a most recent Delta infection	15,280	465	3.0			
	Vaccinated with a most recent Omicron BA.1 infection	26,207	529	2.0			
	Vaccinated with a most recent Omicron BA.2 infection	38,022	201	0.5			
**Ct<30 infection**	Vaccinated without infection	102,484	4,169	3.9			
	Vaccinated with a most recent Pre-Alpha infection	5,790	155	2.6	12,681	328	2.5
	Vaccinated with a most recent Alpha infection	6,891	173	2.4			
	Vaccinated with a most recent Delta infection	15,430	315	2.0			
	Vaccinated with a most recent Omicron BA.1 infection	26,381	355	1.3			
	Vaccinated with a most recent Omicron BA.2 infection	38,098	125	0.3			
**Symptomatic infection**	Vaccinated without infection	104,780	1,873	1.8			
	Vaccinated with a most recent Pre-Alpha infection	5,876	69	1.2	12,864	145	1.1
	Vaccinated with a most recent Alpha infection	6,988	76	1.1			
	Vaccinated with a most recent Delta infection	15,620	125	0.8			
	Vaccinated with a most recent Omicron BA.1 infection	26,604	132	0.5			
	Vaccinated with a most recent Omicron BA.2 infection	38,161	62	0.2			

Supplementary Table 1. Proportion of study assessments with positive PCR results in the correlates of protection analysis. Three outcomes are examined: all infection in the Omicron BA.4/5 epoch; infections with a moderate to high viral load (Ct<30) in the Omicron BA.4/5 epoch; infections with a self-reported symptom in the Omicron BA.4/5 epoch. In the main model, pre-Alpha and Alpha infections were combined because the estimates were similar.

	ChAdOx1-BNT162b2 (N=64940)	ChAdOx1-mRNA-1273 (N=21960)	ChAdOx1-Infection (N=10830)	BNT162b2-BNT162b2 (N=44197)	BNT162b2-mRNA-1273 (N=7248)	BNT162b2-Infection (N=4974)	Total (N=154149)	p value
**Months from second vaccination to booster/infection**								
**Median**	6	6	5	6	6	5	6	
**Q1, Q3**	6, 7	6, 6	4, 6	6, 7	5, 7	4, 6	6, 7	
**Age (years)**								< 0.001
**Median**	63	56	50	66	41	39	60	
**Q1, Q3**	53, 71	49, 62	43, 59	52, 74	34, 60	32, 56	50, 69	
**Sex**								< 0.001
**Female**	34760 (53.5%)	11571 (52.7%)	5757 (53.2%)	25356 (57.4%)	3843 (53.0%)	2793 (56.2%)	84080 (54.5%)	
**Male**	30180 (46.5%)	10389 (47.3%)	5073 (46.8%)	18841 (42.6%)	3405 (47.0%)	2181 (43.8%)	70069 (45.5%)	
**Ethnicity**								< 0.001
**Non-white**	2363 (3.6%)	844 (3.8%)	504 (4.7%)	2043 (4.6%)	351 (4.8%)	332 (6.7%)	6437 (4.2%)	
**White**	62577 (96.4%)	21116 (96.2%)	10326 (95.3%)	42154 (95.4%)	6897 (95.2%)	4642 (93.3%)	147712 (95.8%)	
**Report having a long-term health condition**								
**No**	45834 (70.6%)	17428 (79.4%)	8684 (80.2%)	29429 (66.6%)	5635 (77.7%)	3964 (79.7%)	110974 (72.0%)	
**Yes**	19106 (29.4%)	4532 (20.6%)	2146 (19.8%)	14768 (33.4%)	1613 (22.3%)	1010 (20.3%)	43175 (28.0%)	
**Healthcare worker**								< 0.001
**No**	64184 (98.8%)	21817 (99.3%)	10701 (98.8%)	41365 (93.6%)	7181 (99.1%)	4703 (94.6%)	149951 (97.3%)	
**Yes**	756 (1.2%)	143 (0.7%)	129 (1.2%)	2832 (6.4%)	67 (0.9%)	271 (5.4%)	4198 (2.7%)	
**Infection type**							**Total (N=15804)**	
**Delta**			9696 (89.5%)			3194 (64.3%)	12890 (81.6%)	< 0.001
**Omicron, BA.1**			1119 (10.3%)			1596 (32.1%)	2715 (17.2%)	
**Omicron, BA.2**			13 (0.1%)			180 (3.6%)	193 (1.2%)	
**Other**			2 (0.0%)			0 (0.0%)	2 (0.0%)	
**Missing**			0			4	4	
**Ct value**								< 0.001
**Median**			20			21	20	
**Q1, Q3**			16, 26			17, 26	17, 26	
**Symptom**								< 0.001
**No**			2594 (24.1%)			1451 (29.4%)	4045 (25.8%)	
**Yes, classic symptoms**			4444 (41.3%)			1782 (36.1%)	6226 (39.6%)	
**Yes, other symptoms**			3735 (34.7%)			1701 (34.5%)	5436 (34.6%)	
**Missing**			57			40	97	

Supplementary Table 2. Characteristics of all participants included in the antibody trajectory analyses. Participants were divided into six groups based on the primary vaccine course (ChAdOx1 or BNT162b2) and the third/booster vaccination (BNT162b2 or mRNA-1273) or infection. Continuous variables are compared using Kruskall-Wallis tests, and categorical variables are compared using two-sided Chi-squared tests.

	ChAdOx1-BNT162b2 (N=41152)	ChAdOx1-mRNA-1273 (N=14748)	ChAdOx1-Infection (N=4214)	BNT162b2-BNT162b2 (N=24749)	BNT162b2-mRNA-1273 (N=4403)	BNT162b2-Infection (N=1857)	Total (N=91123)	p value
**Months from second vaccination to booster/infection**								< 0.001
**Median**	6	6	4	6	6	4	6	
**Q1, Q3**	6, 7	6, 6	3, 5	6, 7	5, 7	3, 5	6, 7	
**Age (years)**								< 0.001
**Median**	63	57	50	66	46	39	61	
**Q1, Q3**	55, 71	51, 62	43, 59	55, 74	35, 63	33, 54	52, 70	
**Sex**								< 0.001
**Female**	22213 (54.0%)	7838 (53.1%)	2299 (54.6%)	14145 (57.2%)	2345 (53.3%)	1045 (56.3%)	49885 (54.7%)	
**Male**	18939 (46.0%)	6910 (46.9%)	1915 (45.4%)	10604 (42.8%)	2058 (46.7%)	812 (43.7%)	41238 (45.3%)	
**Ethnicity**								< 0.001
**Non-white**	1249 (3.0%)	507 (3.4%)	200 (4.7%)	958 (3.9%)	179 (4.1%)	121 (6.5%)	3214 (3.5%)	
**White**	39903 (97.0%)	14241 (96.6%)	4014 (95.3%)	23791 (96.1%)	4224 (95.9%)	1736 (93.5%)	87909 (96.5%)	
**Report having a long-term health condition**								< 0.001
**No**	29072 (70.6%)	11662 (79.1%)	3345 (79.4%)	16452 (66.5%)	3360 (76.3%)	1497 (80.6%)	65388 (71.8%)	
**Yes**	12080 (29.4%)	3086 (20.9%)	869 (20.6%)	8297 (33.5%)	1043 (23.7%)	360 (19.4%)	25735 (28.2%)	
**Healthcare worker**								< 0.001
**No**	40725 (99.0%)	14655 (99.4%)	4144 (98.3%)	23379 (94.5%)	4362 (99.1%)	1727 (93.0%)	88992 (97.7%)	
**Yes**	427 (1.0%)	93 (0.6%)	70 (1.7%)	1370 (5.5%)	41 (0.9%)	130 (7.0%)	2131 (2.3%)	
**Infection type**							**Total (N=6071)**	< 0.001
**Delta**			3949 (93.7%)			1198 (64.5%)	5147 (84.8%)	
**Omicron, BA.1**			253 (6.0%)			552 (29.7%)	805 (13.3%)	
**Omicron, BA.2**			10 (0.2%)			106 (5.7%)	116 (1.9%)	
**Other**			2 (0.0%)			0 (0.0%)	2 (0.0%)	
**Missing**			0			1	1	
**Ct value**								< 0.001
**Median**			20			21	20	
**Q1, Q3**			16, 26			17, 26	17, 26	
**Symptom**								< 0.001
**No**			966 (23.0%)			519 (28.1%)	1485 (24.6%)	
**Yes, classic symptoms**			1889 (45.0%)			735 (39.8%)	2624 (43.4%)	
**Yes, other symptoms**			1341 (32.0%)			591 (32.0%)	1932 (32.0%)	
**Missing**			18			12	30	

Supplementary Table 3. Characteristics of participants included in the antibody decline analyses post third/booster vaccination or infection. Participants were divided into six groups based on the primary vaccine course (ChAdOx1 or BNT162b2) and the third/booster vaccination (BNT162b2 or mRNA-1273) or infection. Characteristics in this subgroup are similar to the overall population (**Supplementary Table 2**). Continuous variables are compared using Kruskall-Wallis tests, and categorical variables are compared using two-sided Chi-squared tests.

Age (years)	Primary vaccine	Booster/Infection	Median antibody levels at 42 days (95% Crl) (BAU/mL)	Median half-life (95%Crl) (days)	Median days from booster/infection to reaching 67% protection (95%Crl)
**30**	BNT162b2	mRNA-1273	2144 (1905-2418)	100 (84-125)	102 (55-137)
		Infection	1880 (1625-2180)	130 (103-180)	280 (145-611)
**40**	ChAdOX1	BNT162b2	1441 (1300-1530)	84 (80-99)	56 (0-74)
		mRNA-1273	1940 (1820-2064)	69 (64-74)	83 (63-97)
		Infection	1270 (1119-1435)	85 (74-101)	169 (118-249)
	BNT162b2	BNT162b2	1558 (1482-1638)	95 (88-102)	56 (0-83)
		mRNA-1273	1940 (1774-2121)	101 (88-117)	95 (53-123)
		Infection	1549 (1340-1796)	156 (119-226)	270 (140-563)
**55**	ChAdOX1	BNT162b2	1497 (1345-1572)	78 (75-90)	44 (0-59)
		mRNA-1273	2090 (1995-2190)	63 (61-66)	72 (58-85)
		Infection	1402 (1222-1608)	96 (80-119)	152 (106-233)
	BNT162b2	BNT162b2	1402 (1326-1482)	98 (91-106)	48 (0-65)
		mRNA-1273	2015 (1846-2200)	93 (82-107)	80 (45-106)
		Infection	1261 (1068-1495)	178 (117-382)	243 (123-503)
**70**	ChAdOX1	BNT162b2	1199 (1104-1263)	96 (91-110)	0 (0-0)
		mRNA-1273	1952 (1789-2127)	67 (62-73)	45 (0-65)
		Infection	1429 (1191-1720)	93 (69-142)	120 (74-201)
	BNT162b2	BNT162b2	1250 (1180-1326)	110 (101-121)	0 (0-0)
		mRNA-1273	2430 (2093-2817)	81 (69-98)	51 (0-82)
		Infection	1097 (865-1385)	175 (83-NE)	187 (73-NE)

Supplementary Table 4. Posterior predicted anti-spike IgG levels at 42 days from booster/infection (BAU/mL), half-lives after booster/infection (days), and time from booster/infection to reaching antibody levels associated with 67% protection. Results were separated by age (30, 40, 55, 70y), primary vaccine course (ChAdOx1 or BNT162b2), and booster/third vaccination or infection (BNT162b2, mRNA-1273, infection). Results were estimated at the reference category (female, white ethnicity, 6 months between second vaccination and booster/infection, not reporting a long-term health condition, not working in healthcare). Comparisons are also plotted in **Figure 3**. NE: Not estimable, due to the antibody levels not declining in the posterior median or upper credible interval. 95% credible intervals for days from booster/infection to reaching the 67% threshold level are calculated from posterior simulations from the GAM model estimating correlates of protection and posterior predictions from the Bayesian linear mixed models estimating antibody levels.

	Median duration (days) between second vaccination and booster/infection (IQR)[range]	Selected duration (days) between second vaccination and booster/infection (10th+90th percentile)	Median age (years) (IQR)[range] after using selected duration range	Age spline (years) (10th,50th,90th percentile)	Number of participants (Total/Decline model)
**ChAdOx1-BNT162b2**	189 (183-197) [40-439]	170-210	63 (53-70) [17-85]	45, 60, 75	64940/41165
**ChAdOx1-mRNA-1273**	187 (182-197) [72-437]	170-210	56 (49-62) [17-85]	45, 55, 65	21960/14748
**ChAdOx1-infection**	143 (98-176) [1-440]	60-200	50 (43-59) [17-85]	40, 50, 70	10830/4214
**BNT162b2-BNT162b2**	191 (184-202) [46-475]	150-220	66 (52-74) [16-85]	40, 65, 80	44197/24767
**BNT162b2-mRNA-1273**	180 (147-201) [67-473]	130-220	41 (34-60) [17-85]	30, 40, 70	7248/4408
**BNT162b2-infection**	140 (108-177) [1-483]	70-220	39 (32-55) [16-85]	30, 40, 70	4974/1857

Supplementary Table 5. Settings for each Bayesian linear mixed model. For each group, two separate models are fitted: 1) piecewise model on antibody decline after the second vaccination and subsequent increase after third/booster vaccination or infection; 2) antibody decline 42 days after the third/booster vaccination or infection. Only participants with a time between second vaccination to booster/infection from the 10th to 90th percentile were included in the models to avoid outlier influence. Age splines were included in the models to account for non-linearity in the association between age and antibody response.

Model term	Priors
**Intercept**	normal (10, 2)
**Slope**	normal (0, 0.5)
**Coefficient for change in intercept (age)**	normal (0, 1)
**Coefficient for change in slope (age)**	normal (0, 0.1)
**Coefficient for change in intercept (duration)**	normal (0, 1)
**Coefficient for change in slope (duration)**	normal (0, 0.1)
**Coefficient for change in intercept (sex)**	normal (0, 1)
**Coefficient for change in slope (sex)**	normal (0, 0.1)
**Coefficient for change in intercept (ethnicity)**	normal (0, 1)
**Coefficient for change in slope (ethnicity)**	normal (0, 0.1)
**Coefficient for change in intercept (long-term health condition)**	normal (0, 1)
**Coefficient for change in slope (long-term health condition)**	normal (0, 0.1)
**Coefficient for change in intercept (healthcare worker)**	normal (0, 1)
**Coefficient for change in slope (healthcare worker)**	normal (0, 0.1)
**Random effect SD: intercept**	normal (0, 1)
**Random effect SD: slope**	normal (0, 0.1)
**Random effect intercept & slope covariance**	lkj_corr_cholesky(1)

Supplementary Table 6. Priors used in the Bayesian linear mixed interval-censored models on estimating antibody decline 42 days after third/booster vaccination or infection. Results remain similar when using weaker priors normal(0,2.5) for all coefficients

	ChAdOx1 primary course				BNT162b2 primary course			
**BNT162b2**	**Estimate**	**l-95% CI**	**u-95% CI**	**Rhat**	**Estimate**	**l-95% CI**	**u-95% CI**	**Rhat**
**Population-level effects**								
**Intercept**	10.1833	10.0771	10.2395	1.00	10.3093	10.2538	10.3647	1.01
**T3**	-0.0848	-0.0899	-0.0740	1.02	-0.0741	-0.0793	-0.0690	1.01
**age1**	-0.2053	-0.2760	-0.1368	1.00	-0.4625	-0.5886	-0.3377	1.00
**age2**	-0.4118	-0.4849	-0.3213	1.00	-0.2543	-0.3820	-0.1259	1.00
**Male1**	-0.3397	-0.3863	-0.2692	1.00	0.0048	-0.0551	0.0639	1.01
**ethnicity1**	-0.0139	-0.1193	0.0964	1.01	0.1252	-0.0204	0.2729	1.00
**lthc1**	-0.0454	-0.0963	0.0024	1.01	-0.0357	-0.0977	0.0272	1.00
**hcw1**	-0.0984	-0.3189	0.1162	1.00	-0.4434	-0.6552	-0.2210	1.03
**dur30**	0.2562	0.1458	0.3345	1.01	0.1283	0.0651	0.1908	1.00
**T3:age1**	0.0066	-0.0001	0.0131	1.01	0.0165	0.0054	0.0279	1.00
**T3:age2**	0.0269	0.0192	0.0330	1.00	0.0174	0.0069	0.0280	1.00
**T3:Male1**	0.0022	-0.0052	0.0065	1.00	-0.0071	-0.0124	-0.0019	1.01
**T3:ethnicity1**	0.0066	-0.0035	0.0171	1.01	0.0095	-0.0036	0.0228	1.00
**T3:lthc1**	-0.0025	-0.0067	0.0028	1.00	-0.0022	-0.0077	0.0033	1.00
**T3:hcw1**	0.0034	-0.0163	0.0216	1.01	0.0290	0.0112	0.0461	1.03
**T3:dur30**	-0.0085	-0.0155	0.0033	1.01	0.0030	-0.0027	0.0087	1.00
**Group-level effects**								
**sd(Intercept)**	0.9699	0.0000	1.1859	1.01	1.0346	0.9935	1.0760	1.01
**sd(T3)**	0.0767	0.0679	0.1092	1.01	0.0782	0.0742	0.0823	1.02
**cor(Intercept,T3)**	-0.3021	-0.4485	-0.2519	1.02	-0.3572	-0.4079	-0.3039	1.01
**sigma**	0.5443	0.5039	0.7175	1.01	0.5205	0.5103	0.5307	1.01
**mRNA-1273**	**Estimate**	**l-95% CI**	**u-95% CI**	**Rhat**	**Estimate**	**l-95% CI**	**u-95% CI**	**Rhat**
**Population-level effects**								
**Intercept**	10.5452	10.4897	10.6000	1.00	10.7881	10.6642	10.9161	1.00
**T3**	-0.1055	-0.1106	-0.1005	1.00	-0.0696	-0.0830	-0.0561	1.00
**age1**	0.0611	-0.0169	0.1383	1.00	-0.2368	-0.5039	0.0222	1.00
**age2**	-0.0279	-0.0898	0.0355	1.01	0.2146	0.0129	0.4160	1.00
**Male1**	-0.2279	-0.2794	-0.1739	1.00	0.2616	0.1636	0.3587	1.00
**ethnicity1**	-0.0165	-0.1629	0.1272	1.00	0.1780	-0.0658	0.4158	1.00
**lthc1**	0.0097	-0.0568	0.0758	1.00	0.0375	-0.0866	0.1630	1.00
**hcw1**	-0.1667	-0.4833	0.1602	1.00	0.3072	-0.2513	0.8681	1.00
**dur30**	0.0629	-0.0324	0.1584	1.00	0.0344	-0.0499	0.1184	1.00
**T3:age1**	-0.0088	-0.0160	-0.0015	1.00	-0.0077	-0.0356	0.0208	1.00
**T3:age2**	0.0017	-0.0040	0.0072	1.00	-0.0176	-0.0371	0.0021	1.00
**T3:Male1**	0.0114	0.0065	0.0162	1.00	-0.0043	-0.0143	0.0059	1.00
**T3:ethnicity1**	0.0020	-0.0113	0.0157	1.00	-0.0020	-0.0274	0.0235	1.00
**T3:lthc1**	-0.0107	-0.0169	-0.0046	1.00	-0.0155	-0.0280	-0.0033	1.00
**T3:hcw1**	0.0098	-0.0200	0.0392	1.00	-0.0051	-0.0583	0.0471	1.00
**T3:dur30**	0.0097	0.0013	0.0182	1.00	0.0088	-0.0001	0.0176	1.00
**Group-level effects**								
**sd(Intercept)**	1.1007	1.0681	1.1344	1.00	0.8530	0.7744	0.9298	1.00
**sd(T3)**	0.0650	0.0610	0.0689	1.02	0.0645	0.0545	0.0735	1.00
**cor(Intercept,T3)**	-0.3677	-0.4132	-0.3171	1.01	-0.2529	-0.3892	-0.0780	1.00
**sigma**	0.5614	0.5506	0.5722	1.01	0.6416	0.6158	0.6687	1.00

Infection	Estimate	l-95% CI	u-95% CI	Rhat	Estimate	l-95% CI	u-95% CI	Rhat
**Population-level effects**								
**Intercept**	10.3100	10.1285	10.4874	1.00	10.8769	10.6669	11.0903	1.00
**T3**	-0.0816	-0.0938	-0.0693	1.00	-0.0536	-0.0681	-0.0389	1.00
**age1**	0.2739	0.0385	0.5067	1.00	-0.9581	-1.3349	-0.5763	1.00
**age2**	0.1077	-0.1161	0.3375	1.00	-0.6041	-0.9074	-0.3051	1.00
**Male1**	0.0986	-0.0522	0.2455	1.00	0.2569	0.0511	0.4635	1.00
**ethnicity1**	0.3474	-0.0027	0.6994	1.00	0.0710	-0.3371	0.4738	1.00
**lthc1**	0.0371	-0.1485	0.2202	1.00	-0.1520	-0.4081	0.1055	1.00
**hcw1**	-0.5064	-1.0172	0.0171	1.00	-0.3615	-0.7559	0.0423	1.00
**dur30**	0.2355	0.1720	0.2989	1.00	0.2124	0.1325	0.2922	1.00
**T3:age1**	0.0157	-0.0042	0.0357	1.00	0.0251	-0.0061	0.0565	1.00
**T3:age2**	0.0021	-0.0226	0.0267	1.00	0.0078	-0.0354	0.0505	1.00
**T3:Male1**	-0.0029	-0.0156	0.0095	1.00	-0.0111	-0.0256	0.0039	1.00
**T3:ethnicity1**	-0.0186	-0.0449	0.0073	1.00	0.0140	-0.0160	0.0426	1.00
**T3:lthc1**	-0.0040	-0.0201	0.0126	1.00	-0.0071	-0.0275	0.0131	1.00
**T3:hcw1**	0.0449	0.0045	0.0860	1.00	0.0443	0.0070	0.0811	1.00
**T3:dur30**	-0.0068	-0.0115	-0.0021	1.00	-0.0055	-0.0112	0.0002	1.00
**Group-level effects**								
**sd(Intercept)**	1.6666	1.5770	1.7621	1.00	1.5171	1.4052	1.6374	1.00
**sd(T3)**	0.0555	0.0438	0.0668	1.01	0.0579	0.0463	0.0692	1.00
**cor(Intercept,T3)**	-0.8393	-0.9097	-0.7693	1.01	-0.7104	-0.7924	-0.6161	1.00
**sigma**	0.8476	0.8059	0.8911	1.01	0.7039	0.6596	0.7517	1.00

Supplementary Table 7. Model coefficients and MCMC diagnostics for the Bayesian linear mixed models estimating antibody decline 42 days after the third/booster vaccination or infection.
