# Supplementary material for: Protection against SARS-CoV-2 Omicron BA.4/5 variant following booster vaccination or breakthrough infection in the UK
Source: Nat Commun. 2023 May 16;14:2799. doi: 10.1038/s41467-023-38275-1 (PMC10187514; doi:10.1038/s41467-023-38275-1)
Supplement: Supplementary file 3 — Reporting Summary [file 41467_2023_38275_MOESM3_ESM.pdf]

nature portfolio

Corresponding author(s): David Eyre

Last updated by author(s): Apr 12, 2023

Reporting Summary

Nature Portfolio wishes to improve the reproducibility of the work that we publish. This form provides structure for consistency and transparency in reporting. For further information on Nature Portfolio policies, see our Editorial Policies and the Editorial Policy Checklist.

Statistics

For all statistical analyses, confirm that the following items are present in the figure legend, table legend, main text, or Methods section.

n/a	Confirmed	
☐	☒	The exact sample size (*n*) for each experimental group/condition, given as a discrete number and unit of measurement
☐	☒	A statement on whether measurements were taken from distinct samples or whether the same sample was measured repeatedly
☐	☒	The statistical test(s) used AND whether they are one- or two-sided<br>*Only common tests should be described solely by name; describe more complex techniques in the Methods section.*
☐	☒	A description of all covariates tested
☐	☒	A description of any assumptions or corrections, such as tests of normality and adjustment for multiple comparisons
☐	☒	A full description of the statistical parameters including central tendency (e.g. means) or other basic estimates (e.g. regression coefficient) AND variation (e.g. standard deviation) or associated estimates of uncertainty (e.g. confidence intervals)
☐	☒	For null hypothesis testing, the test statistic (e.g. *F*, *t*, *r*) with confidence intervals, effect sizes, degrees of freedom and *P* value noted<br>*Give P values as exact values whenever suitable.*
☐	☒	For Bayesian analysis, information on the choice of priors and Markov chain Monte Carlo settings
☒	☐	For hierarchical and complex designs, identification of the appropriate level for tests and full reporting of outcomes
☒	☐	Estimates of effect sizes (e.g. Cohen's *d*, Pearson's *r*), indicating how they were calculated

Our web collection on statistics for biologists contains articles on many of the points above.

Software and code

Policy information about availability of computer code

Data collection	The UK's Office for National Statistics (ONS) COVID-19 Infection Survey (CIS) randomly selects private households on a continuous basis from address lists and previous surveys. Individuals were surveyed on their socio-demographic characteristics, behaviours, and vaccination status. Combined nose and throat swabs were taken from all consenting household members for SARS-CoV-2 PCR testing. For a random 10-20% of households, individuals ≥16 years were invited to provide blood samples monthly for serological testing. Household members of participants who tested positive were also invited to provide blood monthly for follow-up visits.<br>De-identified study data were accessed through the Office for National Statistics (ONS) Secure Research Service (SRS). The data available in SRS were prepared and processed using Stata MP 17.
Data analysis	All analyses were performed in R 3.6 using the following packages: tidyverse (version 1.3.0), mgcv (version 1.8-31), cenGAM (version 0.5.3), brms (version 2.14.0), splines (version 3.6.1), ggeffects (version 0.14.3), arsenal (version 3.4.0), cowplot (version 1.1.0), bayesplot (version 1.7.2). A copy of the analysis code is available at https://github.com/jiaweioxford/COVID19_booster_infection.

For manuscripts utilizing custom algorithms or software that are central to the research but not yet described in published literature, software must be made available to editors and reviewers. We strongly encourage code deposition in a community repository (e.g. GitHub). See the Nature Portfolio guidelines for submitting code & software for further information.

Data

Policy information about availability of data

All manuscripts must include a data availability statement. This statement should provide the following information, where applicable:

- Accession codes, unique identifiers, or web links for publicly available datasets
- A description of any restrictions on data availability
- For clinical datasets or third party data, please ensure that the statement adheres to our policy

De-identified study data (including the COVID-19 Infection Survey data and vaccination data from NIMS) are available for access by accredited researchers in the ONS Secure Research Service (SRS) for accredited research purposes under part 5, chapter 5 of the Digital Economy Act 2017. Individuals can apply to be an accredited researcher using the short form on https://researchaccreditationservice.ons.gov.uk/ons/ONS_registration.ofml. Accreditation requires completion of a short free course on accessing the SRS. To request access to data in the SRS, researchers must submit a research project application for accreditation in the Research Accreditation Service (RAS). Research project applications are considered by the project team and the Research Accreditation Panel (RAP) established by the UK Statistics Authority at regular meetings. Project application example guidance and an exemplar of a research project application are available. A complete record of accredited researchers and their projects is published on the UK Statistics Authority website to ensure transparency of access to research data. For further information about accreditation, contact Research.Support@ons.gov.uk or visit the SRS website.

Human research participants

Policy information about studies involving human research participants and Sex and Gender in Research.

Reporting on sex and gender

Participants from private households were randomly selected for enrolment, and sex was based on self-reporting. A total of 84,080 females and 70,069 males were included in the analysis. Models were adjusted for sex.

Population characteristics

Among 154,149 participants, the median age at third/booster vaccination or infection was 60y (Interquartile range [IQR] 50-69), 84,080 (54.5%) participants were female, and 147,712 (95.8%) reported white ethnicity. 43,175 (28.0%) reported having a long-term health condition, and 4,198 (2.7%) were healthcare workers.

Recruitment

The Office for National Statistics (ONS) COVID-19 Infection Survey (CIS) is a large household survey with longitudinal follow-up (ISRCTN21086382, https://www.ndm.ox.ac.uk/covid-19/covid-19-infection-survey/protocol-and-information-sheets) (details in20). The study received ethical approval from the South Central Berkshire B Research Ethics Committee (20/SC/0195). Private households are randomly selected on a continuous basis from address lists and previous surveys to provide a representative sample across the UK. Following verbal agreement to participate, a study worker visited each selected household to take written informed consent for individuals aged 2 years and over. Parents or carers provided consent for those aged 2-15 years; those aged 10-15 years also provided written assent. To reduce the probability that invited individuals/households decide not to respond to the survey they are paid generously to participate in the survey, see https://www.ons.gov.uk/surveys/informationforhouseholdsandindividuals/householdandindividualsurveys/covid19infectionsurveycis/howtotakepart. All participants who completed the enrolment visit was offered a £50 voucher, and one £25 voucher for each further visit. For the current analysis we only included individuals aged 16 years and over.

Nevertheless a certain degree of non-response is inevitable. While certain factors might drive non-response to invitations to participate, adjustment for covariates that may influence selection into the sample ensures that estimates of relative effects are not biased by factors that both influence selection into the sample and the risk of the outcome (model-based inference). Factors that were included in the models are listed below. We cannot exclude the possibility that other unmeasured factors that could influence self-selection into the survey and are not strongly associated with factors already included in the model could bias the result.

Ethics oversight

The study received ethical approval from the South Central Berkshire B Research Ethics Committee (20/SC/0195).

Note that full information on the approval of the study protocol must also be provided in the manuscript.

Field-specific reporting

Please select the one below that is the best fit for your research. If you are not sure, read the appropriate sections before making your selection.

☒ Life sciences ☐ Behavioural & social sciences ☐ Ecological, evolutionary & environmental sciences

For a reference copy of the document with all sections, see nature.com/documents/nr-reporting-summary-flat.pdf

Life sciences study design

All studies must disclose on these points even when the disclosure is negative.

Sample size

154,149 participants aged >18y received two SARS-CoV-2 vaccinations with ChAdOx1 or BNT162b2 followed by a BNT162b2 or mRNA-1273 third/booster vaccination or a breakthrough infection were included. All available data were used for the current study, with the timing of the analysis determined by the duration of follow up available, rather than sample size given the number of participants in the study.

Data exclusions	No available data were excluded from the study.
Replication	All measurements and analytical assays were undertaken once given the scale of the study and cost limitations with 100,000s of assays performed. Serially collected samples from the same participants included demonstrate reproducibility over time.<br>The statistical analyses have been successfully replicated by two individuals.
Randomization	Recruitment randomised - we used data from the UK's Office for National Statistics (ONS) COVID-19 Infection Survey (CIS) (ISRCTN21086382) which randomly selects private households on a continuous basis from address lists and previous surveys conducted by the ONS or the Northern Ireland Statistics and Research Agency to provide a representative sample across the four countries comprising the UK (England, Wales, Northern Ireland, Scotland). No intervention.
Blinding	Not done. This was an observational study with no interventions. Results were returned to participants to support their involvement, but this would not be expected to impact the study findings.

Reporting for specific materials, systems and methods

We require information from authors about some types of materials, experimental systems and methods used in many studies. Here, indicate whether each material, system or method listed is relevant to your study. If you are not sure if a list item applies to your research, read the appropriate section before selecting a response.

Materials & experimental systems

n/a	Involved in the study	
☐	☒	Antibodies
☒	☐	Eukaryotic cell lines
☒	☐	Palaeontology and archaeology
☒	☐	Animals and other organisms
☐	☒	Clinical data
☒	☐	Dual use research of concern

Methods

n/a	Involved in the study	
☒	☐	ChIP-seq
☒	☐	Flow cytometry
☒	☐	MRI-based neuroimaging

Antibodies

Antibodies used	The calibrant (mAb45) provided as part of the Thermo Fisher OmniPATH 384 Combi SARS-CoV-2 IgG ELISA kit is a monoclonal antibody.<br>We calibrated the results of the Thermo Fisher OmniPATH assay into WHO international units (binding antibody unit, BAU/mL) using serial dilutions of National Institute for Biological Standards and Control (NIBSC) Working Standard 21/234.
Validation	Details of the validation of the the Thermo Fisher OmniPATH 384 Combi SARS-CoV-2 IgG ELISA kit are provided in the manufacturer's instructions for use. The assay has also been validated in a head-to-head comparison of similar assays (https://doi.org/10.1016/S1473-3099(20)30634-4), where the sensitivity was 99·1% (95%CI 97·8–99·7) and specificity was 99·0% (98·1–99·5). The assay calibrant, mAb45, is described in https://doi.org/10.1016/10.1016/j.cell.2021.02.032.<br><br>Further validation of the antibodies used was performed by comparing serial dilution series of these antibodies on the test platform, ensuring a sigmoidal dose response that these saturated at the upper limit of detection. Reproducibility between batches and stability over time was also assessed using the same method. Comparisons of the performance of these antibodies on a commercial anti-S antibody detection platform are provided in https://doi.org/10.1016/j.cmi.2021.05.041.

Clinical data

Policy information about clinical studies

All manuscripts should comply with the ICMJE guidelines for publication of clinical research and a completed CONSORT checklist must be included with all submissions.

Clinical trial registration	ISRCTN21086382
Study protocol	https://www.ndm.ox.ac.uk/covid-19/covid-19-infection-survey/protocol-and-information-sheets
Data collection	We used data from the UK's Office for National Statistics (ONS) COVID-19 Infection Survey (CIS) (ISRCTN21086382) from 2 March 2021 to 12 September 2022. The UK's ONS CIS randomly selects private households on a continuous basis from address lists and previous surveys. Individuals were surveyed on their socio-demographic characteristics, behaviours, and vaccination status. Combined nose and throat swabs were taken from all consenting household members for SARS-CoV-2 PCR testing. For a random 10-20% of households, individuals ≥16 years were invited to provide blood samples monthly for serological testing. Household members of participants who tested positive were also invited to provide blood monthly for follow-up visits.
Outcomes	SARS-CoV-2 antibody levels were measured using an ELISA detecting anti-trimeric spike IgG developed by the University of Oxford (Thermo Fisher OmniPATH 384 Combi SARS-CoV-2 IgG ELISA).

Our primary analysis outcomes include:
1) We examined the anti-spike IgG levels after the booster vaccination (BNT162b2 or mRNA-1273) and breakthrough vaccination post second-vaccination, and its relationship with age, sex, ethnicity, long-term health conditions, healthcare worker roles, and days between second vaccination and booster/infection using Bayesian linear mixed models.
2) We examined the correlates of protection using generalised additive models with antibody levels and infections (PCR-positive visits) by vaccination and previous infection status.
3) Based on the above results we estimated the duration of protection from a booster vaccination or breakthrough infection to a level associated with 67% protection.

Our secondary analysis outcomes include:
1) We examined the correlates of protection against infections with Ct value <30 and symptomatic infections.
2) We estimated the proportion of participants above the anti-spike IgG antibody threshold level associated with 67% protection by time from third/booster vaccination or infection.
3) We estimated the median protection level by calendar time among different groups.
4) We examined whether there were effects of SARS-CoV-2 variant (Delta vs Omicron BA.1) on antibody responses after infection.
